# Inhibition of cyclin E1 sensitizes hepatocellular carcinoma cells to regorafenib by mcl-1 suppression

**DOI:** 10.1186/s12964-019-0398-3

**Published:** 2019-07-26

**Authors:** Jianliang Xu, Fei Huang, Zhicheng Yao, Changchang Jia, Zhiyong Xiong, Hao Liang, Nan Lin, Meihai Deng

**Affiliations:** 1 Hepatobilliary Surgery Department, The Third affiliated Hospital of Sun Yat-sen University, No. 600, Tianhe District, Guangzhou, Guangdong China; 2 Anesthesiology Department, The third affiliated hospital of Sun Yat-sen University, Guangzhou, Guangdong China; 3 General surgery, The Third affiliated hospital of Sun Yat-sen University, No. 600, Tianhe District, Guangzhou, 510630 Guangdong China; 4 Cell & Gene therapy center, The Third affiliated Hospital of Sun Yat-sen Uuniversity, Guangzhou, Guangdong China

**Keywords:** Cyclin E1, Hepatocellular carcinoma, Regorafenib, Mcl-1

## Abstract

**Background:**

To clarify the effects of cylcin E1 expression on HCC tumor progression, we studied the expression of cyclin E1 and inhibitory efficacy of regorafenib and sorafenib in HCC cells, and investigated a potential therapy that combines regorafenib treatment with cyclin E1 inhibition.

**Methods:**

Western blotting for caspase-3 and Hoechst 33225 staining was used to measure the expression level of apoptosis-related proteins under drug treatment.

**Results:**

Our results showed that enhanced expression of cyclin E1 after transfection compromised apoptosis in HCC cells induced by regorafenib or sorafenib. Conversely, down-regulation of cyclin E1 gene expression or inhibition of cyclin E1 by the cyclin-dependent kinase (CDK) inhibitors dinaciclib (DIN) or flavopiridol sensitized HCC cells to regorafenib and sorafenib by inducing apoptosis. The expression of Mcl-1, which is modulated by STAT3, plays a key role in regulating the therapeutic effects of CDK inhibitors. Xenograft experiments conducted to test the efficacy of regorafenib combined with DIN showed dramatic tumor inhibitory effects due to induction of apoptosis. Our results suggested that the level of cyclin E1 expression in HCCs may be used as a pharmacodynamic biomarker to assess the antitumor effects of regorafenib or sorafenib.

**Conclusions:**

Combining regorafenib and CDK inhibitors may enhance the clinical efficiency of the treatment of HCCs.

**Electronic supplementary material:**

The online version of this article (10.1186/s12964-019-0398-3) contains supplementary material, which is available to authorized users.

## Background

Hepatocellular carcinoma (HCC) is reported to be one of the top three tumors responsible for the global cancer fatalities. Only 30% of patients are diagnosed in the early stages of the disease and able to accept tumor resection or liver transplantation [1]. Patients diagnosed at earlier disease stages have a good chance of survival, since several potential curative treatments are available, such as loco-regional radiofrequency ablation, liver resection, and liver transplantation for patients with portal hypertension and cirrhosis. However, for patients with advanced and/or metastatic cases, treatment choices are quite limited and prognosis is poor [2]. It has been reported that regorafenib and sorafenib inhibit several kinases, including BRAF, CRAF, PDGFR, VEGFRs, and c-Kit [3, 4]. Recent studies have shown that, as pan-kinase inhibitors, sorafenib and regorafenib increase the overall rate of cancer patients and have been used in the treatment of advanced HCC [5]. Their tumor inhibitory effects are correlated with apoptosis induction, cell proliferation inhibition, and tumor angiogenesis suppression [6, 7]. Many apoptosis regulators, including survivin and Mcl-1, modulate the anticancer effects of sorafenib and regorafenib in tumor cells [7, 8]. The success of clinical therapies greatly relies on the discovery of apoptosis pathways and modulators [9].

Uncontrolled cell proliferation in which the cell cycle phases are disrupted is a mark of tumor cells. Upregulation of cyclin family proteins is usually correlated with late stages and poor outcomes in several kinds of tumors, including HCC [10, 11]. Although cyclin-dependent kinase (CDK) inhibitors and other molecular agents that regulate the cell cycle have been widely studied, the role of these agents in tumor therapy was unclear until a CDK inhibitor, palbociclib, was shown to extend the life span of breast cancer patients who were treated with hormones [12, 13]. Data from both preclinical and clinical studies indicate that the combination of cell-cycle regulators and current anticancer treatments may improve tumor treatment efficacy [14]. Sorafenib can downregulate the expression of E2F1, cyclin D (CCND), cyclin E1 (CCNE1), and CDKs, which are the main regulators in cell-cycle pathways. The suppression of cell cycle regulator levels by sorafenib may enhance its antitumor efficacy [7, 15]. It was reported that the expression of cyclin E1 in HCCs was correlated with their response to sorafenib treatment [15], and inhibition of cyclin E1 can sensitize HCCs to sorafenib induced apoptosis [16]. However, it is still unclear whether the expression of cyclin E1 has similar effects on regorafenib sensitivity in HCC.

According to our study, cyclin E1 expression levels were more correlated to the survival of HCC patients and the drug sensitivity of regorafenib and sorafenib, than were CCNA1 or CCND1. Our data suggested that the inhibition of cyclin E1 by dinaciclib (DIN) or flavopiridol (FLA) can suppress HCC cell growth by triggering apoptosis, and enhance the killing effect of regorafenib or sorafenib both in vitro and in vivo.

## Materials and methods

### HCC cell lines and reagents

Several HCC lines were used in our study, HepG2, Hep3B, SK-Hep1, SNU398, and SNU475 were from the American Type Culture Collection, while the Huh-7 cell line was purchased from the Health Science Research Resources Bank. HepG2, Hep3B, and Huh7 were grown in Dulbecco’s modified Eagle’s medium (Life Technologies) supplemented with 10% fetal calf serum and 1% antibiotic–antimycotic solution (Gibco, Paisley, SCT). All other cell lines were cultured in basal RPMI medium (Gibco, Paisley, SCT) with 10% fetal calf serum and 1% antibiotic–antimycotic solution at 37 °C in a 5% CO_2_ incubator. This study has been approved by the Ethics Committee of the third affiliated hospital of Sun yat-sen university.

All the chemicals, including FLA, DIN, regorafenib, and sorafenib, were provided by Selleck (Houston, Texas, US). All drugs were dissolved in dimethyl sulfoxide (DMSO), and stored at − 80 °C in small aliquots. Final DMSO concentrations were less than 0.1%.

### Database analysis

The University of California Santa Cruz Cancer Genomics Browser at https://xenabrowser.net/ was used to analyze *CCNA1, CCND1*, and *CCNE1* gene expression data from The Cancer Genome Atlas (TCGA) database. The plotter program at http://kmplot.com/analysis/ was used to generate Kaplan–Meier curves.

### Cell metabolic activity, cell-cycle distribution, and cell death assays

Cell viability was measured with an MTT [3-(4, 5-dimethylthiazol-2-yl)-2, 5- diphenyltetrazolium bromide] assay (Promega, Beijing, China) as described in the main text. Cell cycle and apoptosis after drug treatment were detected by Annexin-V/Propidium iodide (PI) staining (Thermo Fisher Scientific, Shanghai, China) and flow cytometry, as described in our previous publication [17]. Western blotting for caspase-3 (Cell signaling, Shanghai, China) was used to measure the expression level of apoptosis-related proteins under drug treatment.

### Plasmid and siRNA transfection

Lipofectamine 2000 (Invitrogen, Shanghai, China) mediated plasmid or siRNA transfection was used to manipulate the expression of cyclin E1 and Mcl-1. The siRNA for cyclin E1 was obtained from Santa Cruz. The plasmid expressing cyclin E1 was produced via insertion of cDNA into the pcDNA3.1-HA vector (Addgene, Cambridge, MA, USA). The plasmids for Mcl-1 (#25375), and STAT3 (#74433) were purchased from Addgene. Western blotting was performed to detect the efficacy of gene overexpression or knockdown.

### Immunoblotting

Samples used for this assay were collected from whole-cell lysates. A coomassie assay (Pierce, Rockford, IL) was used to quantify the total protein concentration. Identical amounts of protein were run on sodium dodecyl sulfate polyacrylamide gel electrophoresis gels and electro-transferred to polyvinylidene fluoride membranes. The following primary antibodies were used in this study: Bim, Noxa, PUMA (ProSci, Poway, CA); Mcl-1, caspase 3, and caspase 8 (BD Biosciences); Bcl-2 (DAKO, Carpinteria, CA); cyclin E1 (BD Biosciences), Bax (R&D Systems), Actin (Santa Cruz Biotechnology), Bcl-xL, Bad, cyclin D1, cyclin A1, STAT3, and phosphor-STAT3 (Cell Signaling Technology).

### Reverse transcription-PCR

Cellular RNA was isolated using Trizol (provided by Invitrogen, Rockville, MD) following the manufacturer’s protocol. The RT-PCR reaction mixture contained 1 μg RNA and reverse transcriptase (Promega) with β-actin as the internal control.

List of 5′ and 3′ primers for RT-PCR:

β-actin:

5′-CTTAATGTCACGCACGATTTC-3′.

5′-ACGTTATGGTGATGATATCG-3′.

Mcl-1:

5′-CCGTCCAGCTCCTCTTCG-3′.

5′-CGGACTCAACCTCTACTGTGG-3′.

### Chromatin immunoprecipitation (ChIP) assay and luciferase assay

ChIP (Cell Signaling Technology) was used to analyze the binding efficiency of STAT3 to the Mcl-1 promoter with and without Din treatment. In brief, cells were treated with formaldehyde (1%) at 37 °C for 10 min, harvested in lysis buffer and incubated on ice for another 10 min. Micrococcal nuclease was added to digest the nuclei. After sonication and high-speed centrifugation, chromatin samples were incubated with either STAT3 antibody or the negative control (rabbit serum) at 4 °C overnight. The chromatin was then mixed with protein G beads, and incubated on a rotation bed for 2 h. Protein-DNA complexes that bind to the antibody were eluted and analyzed by PCR.

List of 5′ and 3′ primers for the ChIP assay:

5′-TAGGTGCCGTGCGCAACCCT-3′.

5′-ACTGGAAGGAAGCGGAAGTGAGAA-3′.

The Mcl-1 promoter luciferase reporter assay conducted as previous described by using pGL2-Mcl-1, which was purchased from Addgene (#19132) [18]. Transfection efficiency was normalized by expression of a CMV–β-galactosidase reporter gene (Addgene, #8387).

### Tumor xenograft experiments

All proposals for xenograft operations were reviewed and granted by the Institutional Animal Care and Use Committee of the third affiliated hospital of Sun yat-sen university. All animal operations and postoperative animal treatment were carried out in accordance with the Care and Use of Laboratory Animals Guide published by the NAS and NIH. Huh-7 cells were inoculated into BALB/c athymic (nu^+^/nu^+^) male mice subcutaneously. Mice were then administered regorafenib (20 mg/kg) every day via oral gavage, and/or Din (30 mg/kg) every other day by intraperitoneal injection. Both sorafenib and Din were dissolved in Cremephor EL/95% ethanol (50:50) as a 4X stock solution, and diluted to the final concentration with sterile water before use. Tumor volume was measured every 3 days. Following drug treatment, we excised tumor tissues, which were collected for terminal deoxynucleotidyl transferase dUTP nick end labeling (TUNEL) assays and western blotting.

### Statistical analysis

All the assays in this study consisted of at least three independent sets of experiments. All data are presented as mean ± SD. Differences between two groups were tested using Student’s *t*-test and ANOVA. *P* values < 0.05 were considered statistically significant. The Kaplan–Meier method was used to calculate survival time, and comparisons between animal groups were analyzed using a log-rank test.

## Results

### High cyclin E1 expression is correlated with poor outcomes in HCC

**Fig. 1:**
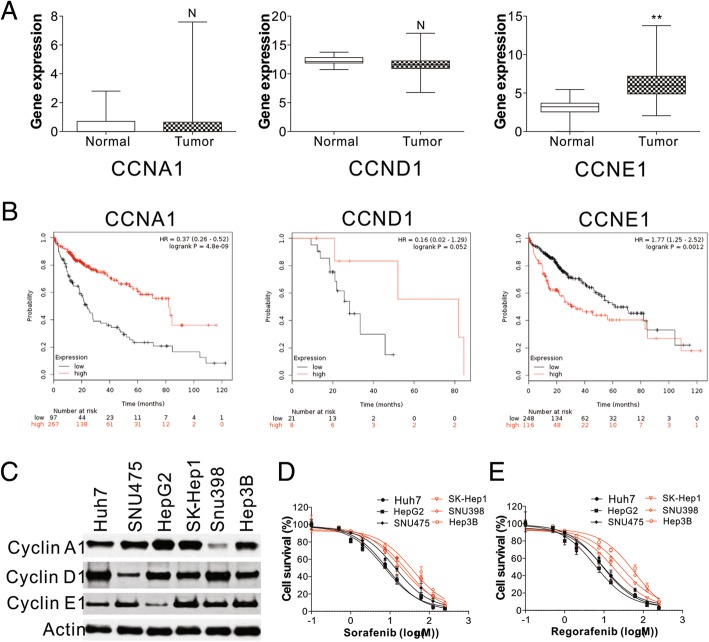
High expression of CCNE1 is related to poor hepatocellular carcinoma outcomes. **a** mRNA expression of CCNA1, CCND1, and CCNE1 in the TCGA liver cancer database. **b** Overall survival (OS) rates of HCC patients with various levels of CCNA1, CCND1, and CCNE1 expression. **c** Expression levels of CCNA1, CCND1, and CCNE1 in the indicated HCC cell lines. **d** The cell viability of the indicated HCC cells treated with various concentrations of sorafenib. **e** The cell viability of the indicated HCC cells treated with various concentrations of regorafenib. The western blots were repeated for 3 times, and representative data were shown. *N* = 3 for D and E. N, *P* > 0.05, **, *P* < 0.05s

Cell-cycle dysregulation is a hallmark of tumor cells [19]. To further study its role in liver malignancy, we examined the expression of several cyclin family members in large scale cancer datasets provided by TCGA, including CCNA1, CCND1, and CCNE1. TCGA RNA Seq data showed significant upregulation of CCNE1 in 371 liver tumors, compared to 50 normal control tissues (Fig. 1a). In contrast, there was no difference in CCNA1 or CCND1 expression in liver tumors and normal control tissues (Fig. 1a). Importantly, a significant correlation was found between high cyclin E1 expression and poor survival rates of HCC patients (HR = 1.77; *P* = 0.0012) (Fig. 1b). Conversely, high expression of CCNA1 and CCND1 benefited HCC patients, resulting in better survival outcomes (Fig. 1b). The survival of cancer patients is largely dependent on their response to drug therapy. Regorafenib and sorafenib are two drugs commonly used to treat HCC. We therefore analyzed the relationship between cyclin E1 expression and the killing effect of regorafenib and sorafenib. By analyzing 6 different HCC cell lines, we found that cyclin E1 expression was lower in Huh7, HepG2, SNU475, but higher in SK-Hep1, SNU398, and Hep3B cells (Fig. 1c). The cell lines with lower levels of cyclin E1 expression had better responses to sorafenib or regorafenib treatment, since their IC50 values were lower than the 3 cell lines with higher cyclin E1 expression (Fig. 1d, e, Additional file 1: Figure S1). Our results, together with the correlation of cyclin E1 expression in HCC, highly suggest that cyclin E1 expression plays an important role in HCC therapy.

### Enhanced expression of cyclin E1 suppresses drug sensitivity

**Fig. 2:**
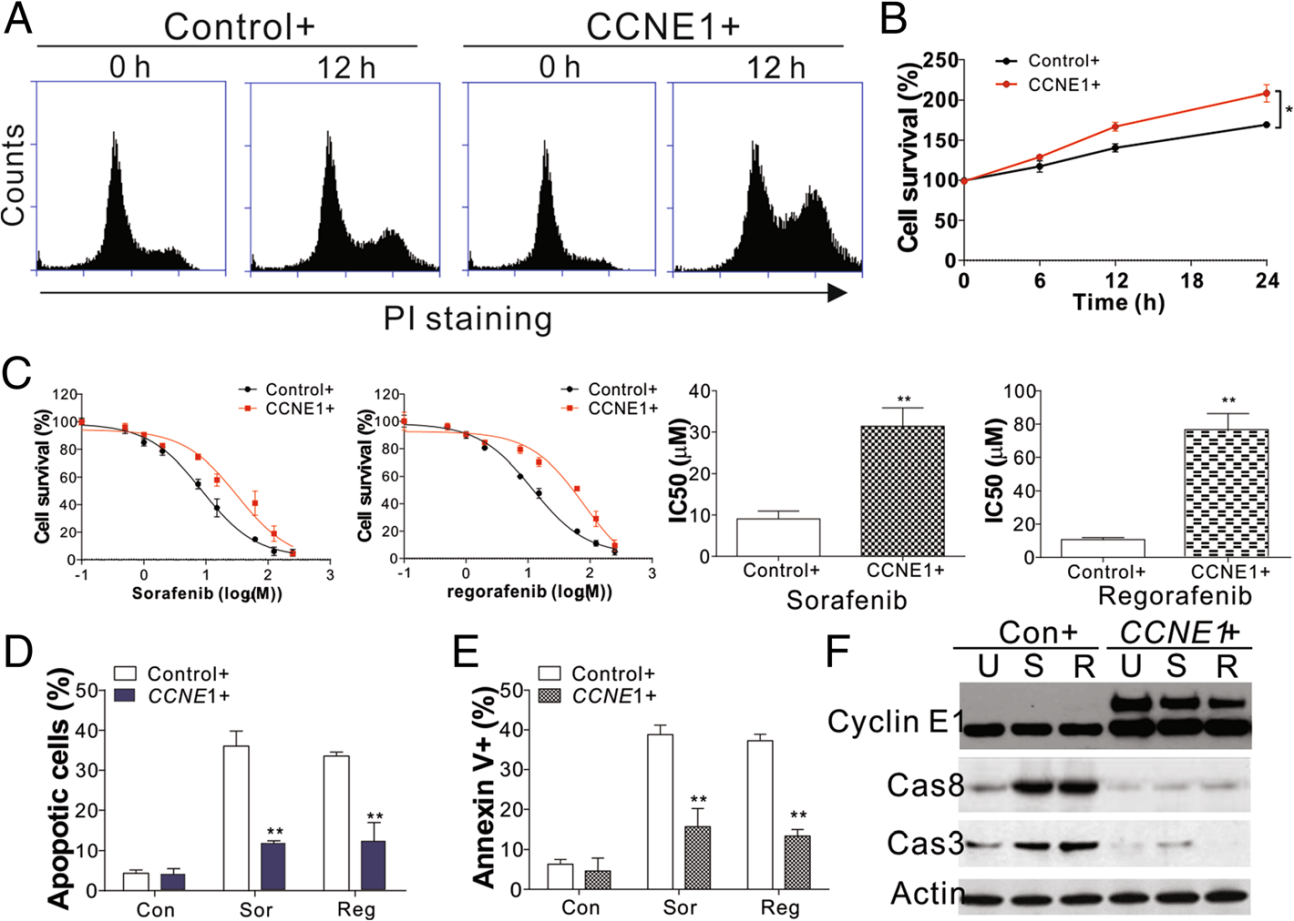
CCNE1 expression suppressed sorafenib and regorafenib induced cell death. **a** The cell cycle of Huh7 cells transfected with the control or CCNE1 plasmid. **b** The cell viability of Huh7 cells transfected with the control or CCNE1 plasmid. **c** The cell viability of Huh7 cells transfected with the control or CCNE1 plasmid and treated with different concentrations of regorafenib or sorafenib. **d** Hoechst 33258 staining for apoptosis of Huh7 cells transfected with the control or CCNE1 plasmid and treated with 8 μM regorafenib or 5 μM sorafenib. **f** Annexin V/PI staining for apoptosis of Huh7 cells transfected with the control or CCNE1 plasmid and treated with 8 μM regorafenib or 5 μM sorafenib. **f** Western blot of CCNE1 and cleaved caspase 3/8 in Huh7 cells transfected with the control or CCNE1 plasmid and treated with 8 μM regorafenib or 5 μM sorafenib. The western blots and flow cytometry were repeated for 3 times, and representative data were shown. *N* = 3 for **b**-**e**. *, *P* < 0.05, **, *P* < 0.05

Cyclin E1 forms a functional kinase complex with CDK2 at the G1/S boundary to regulate cell cycle progression into S phase [20]. We found that the increased expression of cyclin E1 in Huh7 and HepG2 cells accelerated the cell cycle by promoting the G1/S phase (Fig. 2a), and therefore increased cell proliferation (Fig. 2b, Additional file 2:Figure S2A). Previous studies suggested that the expression of cyclin E1 is negatively associated with anti-cancer drug sensitivity [16]. To further examine the modulatory effects of cyclin E1 on anti-cancer sensitivity, we measured the IC_50_ of regorafenib and sorafenib after cyclin E1 overexpression. Cyclin E1 overexpression significantly reduced sensitivity to sorafenib and regorafenib in Huh7 HCC cells (Fig. 2c). Apoptosis is an important mechanism for the tumor inhibitory effects of regorafenib and sorafenib [6, 16]. We found that cyclin E1 overexpression suppressed the effects of regorafenib and sorafenib, which elicited apoptosis in HCC cells (Fig. 2d-f, Additional file 2: Figure S2B, C). This demonstrated that the tumor inhibitory efficacy of regorafenib and sorafenib is mainly mediated by cyclin E1 expression in HCC cells.

### The antitumor activity of sorafenib and regorafenib is enhanced by cyclin E1 inhibition

**Fig. 3:**
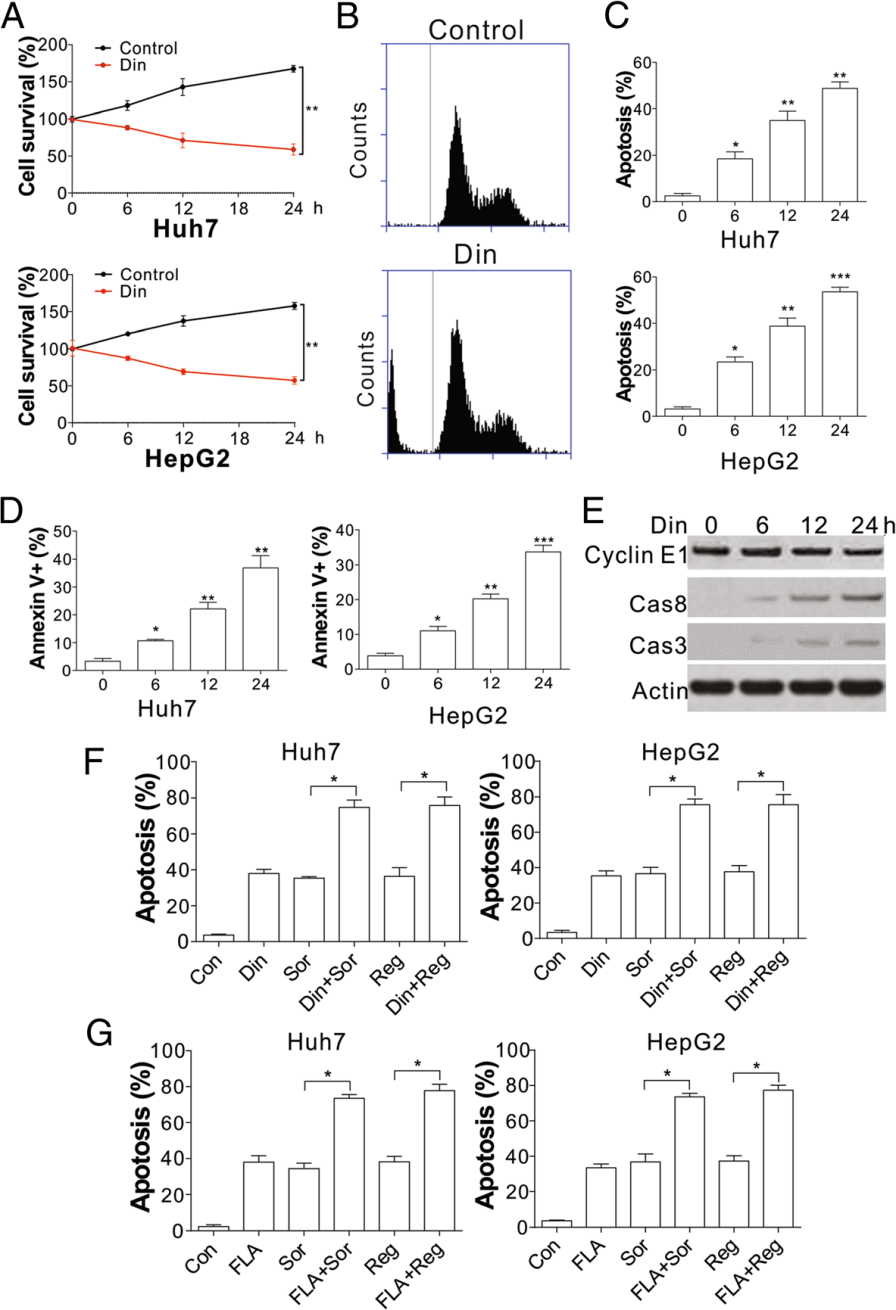
Inhibition of CCNE1 sensitized hepatocellular carcinoma cells to regorafenib and sorafenib induced apoptosis. **a** The cell viability of Huh7 and HepG2 cells treated with 50 nM Din. **b** The cell cycle of Huh7 cells treated with 50 nM Din. **c** Hoechst 33258 staining for apoptosis of Huh7 and HepG2 cells treated with 50 nM Din at the indicated time points. **d** Annexin V/PI staining for apoptosis of Huh7 and HepG2 cells treated with 50 nM Din at the indicated time points. **e** Western blot of cyclin E1, and cleaved caspase 3/8 in Huh7 cells treated with 50 nM Din at indicated the time points. **f** Hoechst 33258 staining for apoptosis of Huh7 and HepG2 cells treated with 50 nM Din in combination with 8 μM regorafenib or 5 μM sorafenib. **g** Hoechst 33258 staining for apoptosis of Huh7 and HepG2 cells treated with 100 nM FLA in combination with 8 μM regorafenib or 5 μM sorafenib. The western blots and flow cytometry were repeated for 3 times, and representative data were shown. *N* = 3 for **a**, **c**, **d**, **f**, **g**. *, *P* < 0.05, **, P < 0.05, ***, *P* < 0.001

In the next step, we tested whether inhibition of cyclin E1 reduces HCC survival and increases sensitivity to regorafenib or sorafenib treatment. Din was reported to suppress cyclin E1 and exert potent apoptotic and antitumor effects in multiple cancers [21]. We therefore treated HCC cells with Din. We found that Din treatment not only suppressed HCC proliferation, but also led to cell loss (Fig. 3a). Cell cycle analysis with flow cytometry revealed that Din treatment elicited G1/S arrest (Fig. 3b), and a higher ratio of hypodiploid cells, indicating apoptosis (Fig. 3b). We therefore analyzed the apoptotic signal by Hoechst and annexin-V/PI staining and found that Din induced HCC cell death in a time-dependent manner (Fig. 3c, d). Furthermore, Din treatment also resulted in the cleavage of caspase-8 and caspase-3 (Fig. 3e), suggesting that Din treatment led to HCC apoptosis. However, Din treatment did not obviously change the expression of cyclin E1 (Fig. 3e). Furthermore, when the combination of sorafenib or regorafenib and Din was examined in vitro to test whether their tumor inhibitory effects were enhanced. We found that sorafenib or regorafenib induced apoptosis was markedly increased by Din in HepG2 and Huh7 HCC cells (Fig. 3f). To exclude the off-target effects of Din, we also used other cyclin E1 inhibitors, FLA, and cyclin E1 siRNA. As with Din, the combination of sorafenib or regorafenib with FLA induced higher apoptosis in Huh7 and HepG2 cells (Fig. 3g). Although depletion of cyclin E1 by siRNA did not elicit cell death in Huh7 cells (Additional file 3: Figure S3A), it suppressed cell proliferation (Additional file 3: Figure S3A), and sensitized the Huh7 cells to sorafenib or regorafenib induced apoptosis (Additional file 3: Figure S3B, C). Consistently, Din or FLA treatment also enhanced the killing effects of sorafenib or regorafenib in SK-HEP-1 cells, which has high cyclin E1 expression, and showed resistant to regorafenib and sorafenib (Additional file 3: Figure S3D). Therefore, our data suggested that the killing effects of regorafenib and sorafenib were promoted by inhibition or depletion of cyclin E1 in HCC cells.

### Mcl-1 mediates the apoptosis induced by cyclin E1 inhibition

**Fig. 4:**
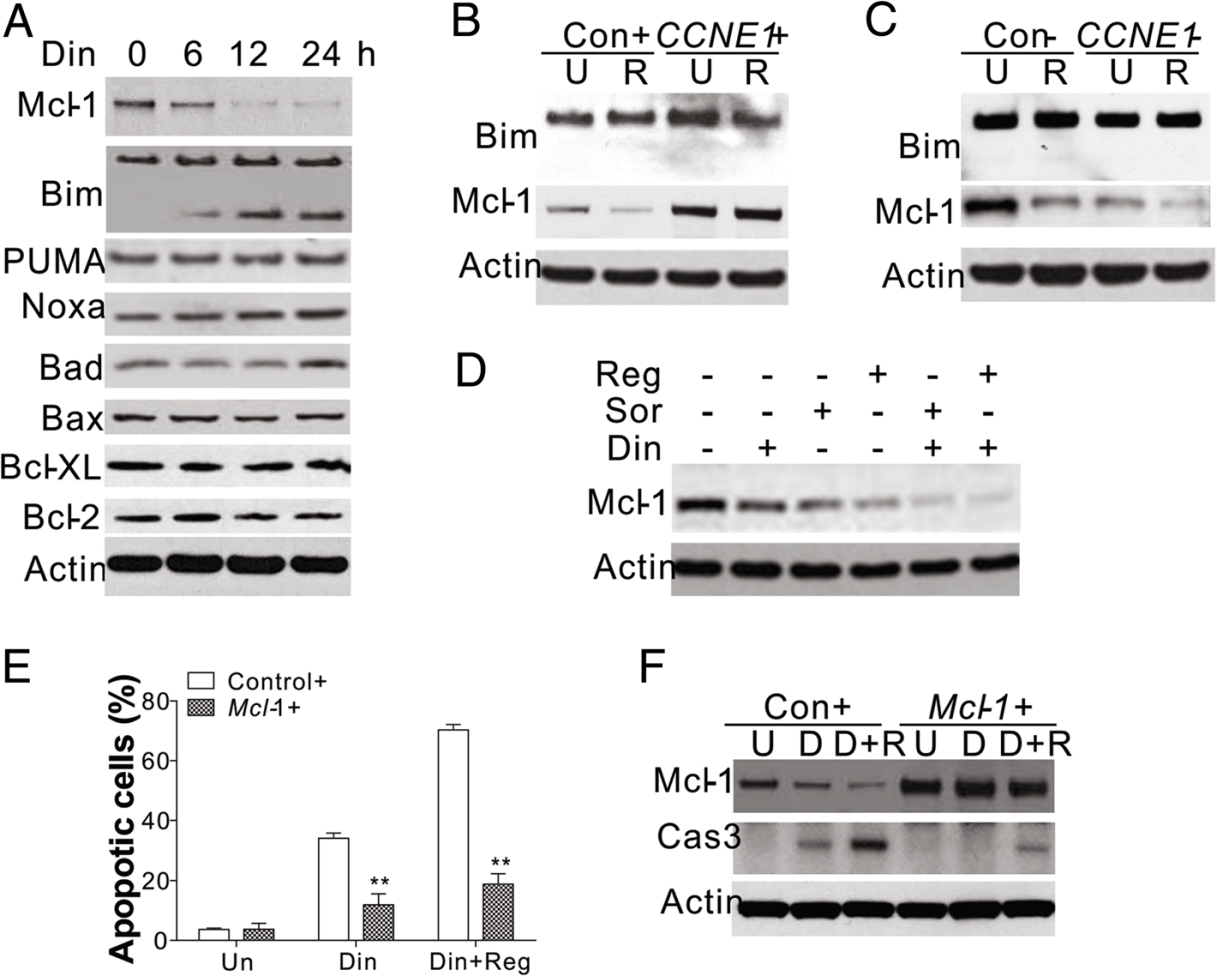
Inhibition of CCNE1 induced HCC apoptosis by targeting Mcl-1. **a** The expression of the target proteins in Huh7 cells treated with 50 nM Din at the specified time points. **b** The expression of Bim and Mcl-1 in Huh7 cells transfected with the CCNE1 or control plasmid and treated with 8 μM regorafenib. **c** The expression of Bim and Mcl-1 in Huh7 cells transfected with CCNE1 or control siRNA and treated with 8 μM regorafenib. **d** The expression of Mcl-1 in Huh7 cells treated with 50 nM Din in combination with 8 μM regorafenib or 5 μM sorafenib. **e** Hoechst 33258 staining for apoptosis of Huh7 cells transfected with the control or Mcl-1 plasmid and treated with 50 nM Din in combination with 8 μM regorafenib. **f** The expression of cleaved caspase-3 and Mcl-1 in Huh7 cells transfected with the control or Mcl-1 plasmid and treated with 50 nM Din in combination with 8 μM regorafenib. The western blots were repeated for 3 times, and representative data were shown. *N* = 3 for **e**. *, *P* < 0.05, **, *P* < 0.05

Bcl-2 homologs are vital for apoptosis. Therefore, we studied the change in the expression of Bcl-2 homologs in response to Din treatment. We found that Din treatment suppressed the expression of Mcl-1 in Huh7 cells, and increased cleavage of Bim (Fig. 4a). In contrast, Din treatment did not significantly change the expression of other Bcl-2 family proteins, including PUMA, Noxa, Bad, Bax, Bcl-XL, or Bcl-2 (Fig. 4a). Inhibition of Mcl-1 expression in Huh7 cells treated with regorafenib, was relieved by cyclin E1 overexpression (Fig. 4b), but enhanced by cyclin E1 knockdown (Fig. 4c). However, regorafenib treatment did not change the expression or cleavage of Bim, which was also not affected by changes in cyclin E1 expression (Fig. 4b, c). Furthermore, the combination of Din and regorafenib enhanced the suppression of Mcl-1 (Fig. 4d), which was correlated with a synergic effect on apoptosis. This suggested that Din enhanced the killing effect of regorafenib by inhibiting the expression of Mcl-1. To confirm the role of Mcl-1 in mediating Din induced cell death, we overexpressed Mcl-1 in Huh7 cells. Our results showed that the apoptosis induced by Din was reversed by Mcl-1 overexpression (Fig. 4e, f). Furthermore, upregulation of Mcl-1 expression also abolished the synergic effect of Din and regorafenib or sorafenib (Fig. 4e, f, Additional file 4: Figure S4A, and B). These results suggest that the antitumor effects of regorafenib can be enhanced by FLA and that cyclin E1 and Mcl-1 are the key molecules mediating this enhancement of tumor inhibition.

### Cyclin E1 promotes mcl-1 expression by STAT3

**Fig. 5:**
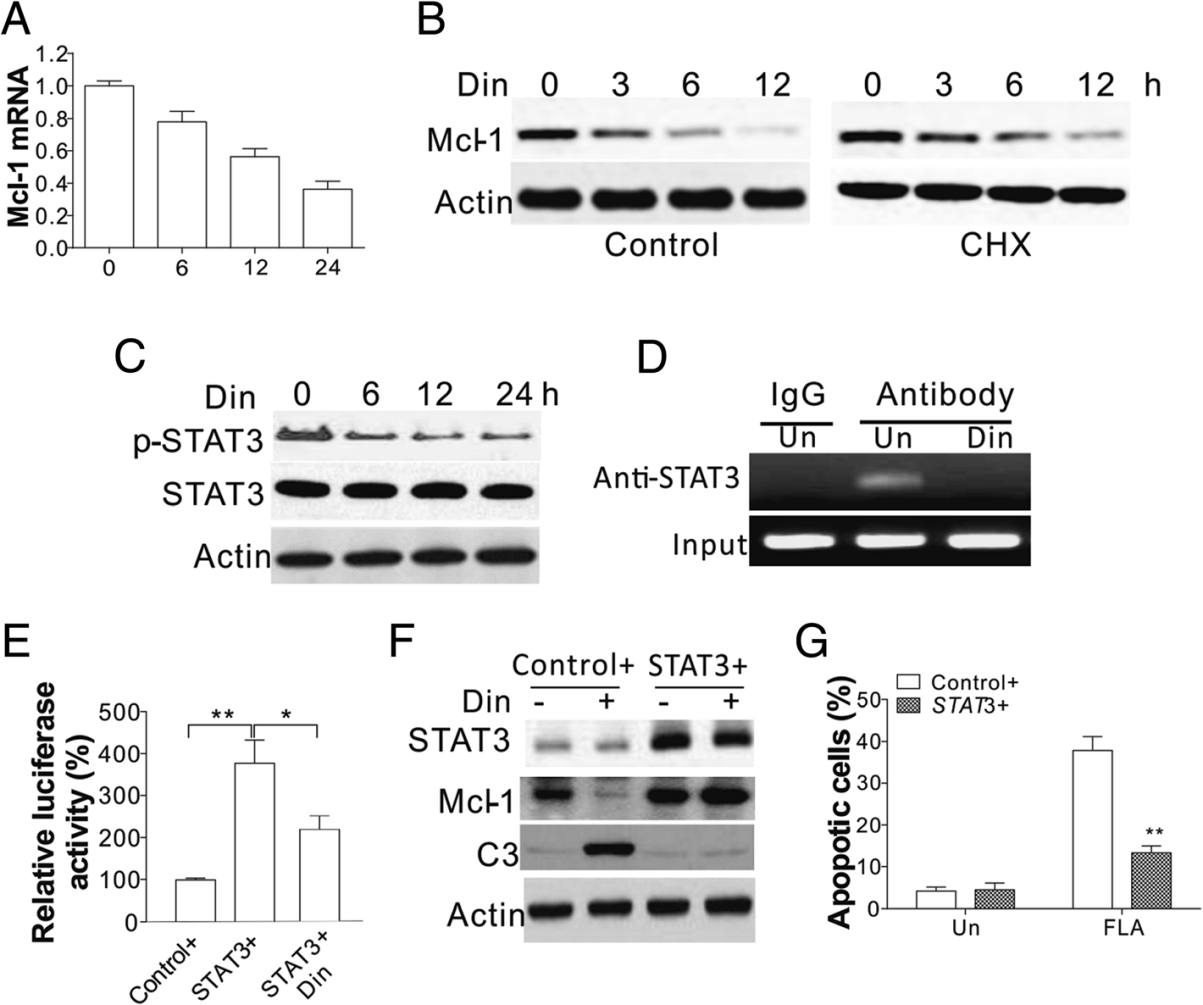
Din mediated the downregulation of Mcl-1 by suppressing transcription of STAT3. **a** The levels of Mcl-1 mRNA in Huh7 cells after treatment with 50 nM Din at the indicated time points. **b** Mcl-1 protein levels in Huh7 cells treated with 50 nM Din with or without pretreatment of 1 μg/ml CHX. **c** The expression of p-STAT3, and total STAT3 in Huh7 cells treated with 50 nM Din at different time points. **d** The binding of STAT3 to Mcl-1 proteins in Huh7 cells and treated with 50 nM Din for 24 h. **e** The luciferase reporter activity of pGL-Mcl-1 reporter in Huh7 cells transfected with control or STAT3 plasmids, followed by 50 nM Din treatment. **f** Expression of STAT3, cleaved caspase-3, and Mcl-1 in Huh7 cells transfected with the control or STAT3 plasmid and treated with 50 nM Din. **g** Hoechst 33258 staining for apoptosis of Huh7 cells transfected with the control or STAT3 plasmid and treated with 50 nM Din. The western blots were repeated for 3 times, and representative data were shown. *N* = 3 for **a**, **e**, **g**. **, *P* < 0.05

It has been reported that Mcl-1 can be controlled at both the transcriptional and post-transcriptional levels [16, 22]. We firstly checked changes in Mcl-1 transcription in response to Din treatment, and found that inhibition of cyclin E1 suppressed Mcl-1 mRNA levels (Fig. 5a). In contrast, pre-treatment with cycloheximide (CHX) did not accelerate the downregulation of Mcl-1 by Din (Fig. 5b), suggesting that Din inhibited the Mcl-1 expression at the mRNA level rather than by protein degradation. STAT3 is significant in the Mcl-1 response to cyclin E1 inhibition [23]. We found that Din treatment inhibited the activation of STAT3 in a time dependent manner (Fig. 5c). Additionally, Din treatment also abrogated the binding of STAT3 to the Mcl-1 promoter (Fig. 5d), suggesting that STAT3 is the upstream transcription factor acting in response to cyclin E1 inhibition. Enhanced expression of STAT3 increased the Mcl-1 luciferase reporter activity, which was suppressed by Din treatment (Fig. 5e), further suggesting the role of STAT3 in Mcl-1 transcription. Consistent with this, overexpression of STAT3 in Huh7 cells restored the expression of Mcl-1 (Fig. 5f), and therefore suppressed Din induced apoptosis (Fig. 5g). Collectively, our results suggested that inhibition of cyclin E1 suppressed Mcl-1 expression by inhibiting transcription through STAT3.

### Din enhanced the killing effect of regorafenib in vivo

**Fig. 6:**
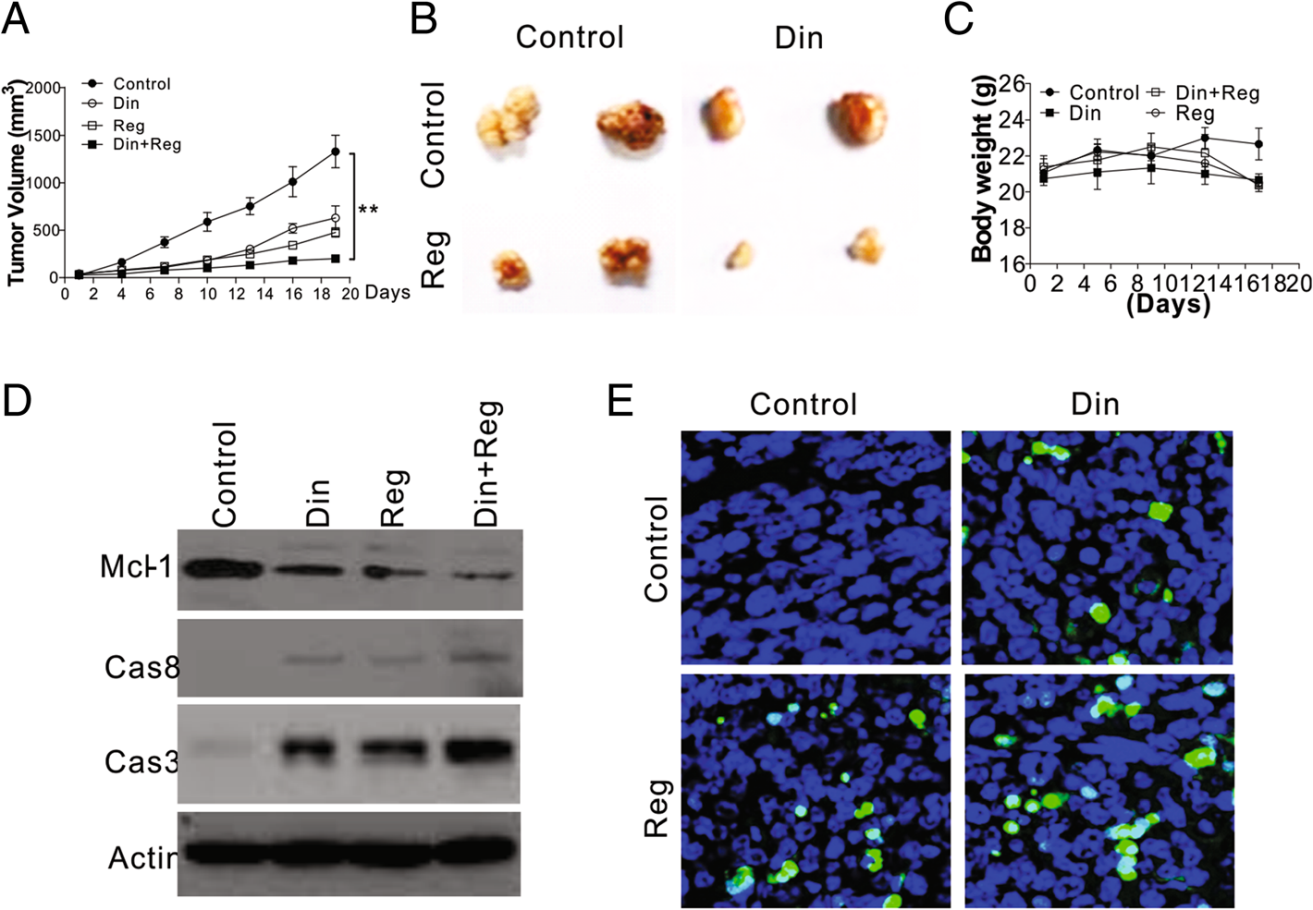
Din enhanced the tumor inhibitory effect of regorafenib in vivo. Male BALB/c athymic knockout mice were subcutaneously inoculated with Huh-7 cells. They were then administered regorafenib (20 mg/kg/d), with and without Din (30 mg/kg, every other day). **a** Tumor size difference (for each group, *n* = 5). **b** Representative tumors from each group. **c** The body weight of mice in each treatment groups. **d** The expression of cleaved caspase 3/8 and Mcl-1 in the tumors from the different groups. **e** Changes in tumor cell apoptosis (TUNEL assay) after drug treatment. The western blots and immunostaining were repeated for 3 times, and representative data were shown. **, *P* < 0.05

We next examined the effect of the combination of regorafenib and cyclin E1 on the growth of Huh7 in vivo. We implanted Huh7 cells subcutaneously into the flanks of BALB/c nude mice. After 10 days, when the size of the tumors reached 100 mm^3^, the mice used in our study were randomly divided into 4 groups and administered regorafenib, Din, regorafenib combined with Din, or PBS as a negative control (Control) by intraperitoneal injection. After a further 19 days, we found that both regorafenib and Din suppressed the tumor growth (Fig. 6a, b). The combination of regorafenib and Din suppressed the growth of tumor significantly more than either regorafenib or Din alone (Fig. 6a, b). The single treatment or combination of drugs did not obviously change the body weight of mice, ruling out their side effects (Fig. 6c). We also analyzed the activity of caspase 3 and 8 in different tumors after treatment, and found that the combination of regorafenib and Din had higher caspase-3/8 activation in Huh7 xenografts (Fig. 6d). The expression of Mcl-1 was also suppressed in regorafenib and Din treated tumors (Fig. 6d). TUNEL staining indicated that the combination treatment maximized apoptosis in Huh7 tumors (Fig. 6e). Therefore, inhibition of cyclin E1 enhances the therapeutic effect of regorafenib in vivo.

## Discussion

Cyclin E1 drives cell proliferation by initiating DNA replication and activating CDK2. Together with cyclin D/CDK4, cyclin E/CDK2 phosphorylates RB to activate the genes downstream of E2F and promotes the transition from G1 to S phase [11]. Higher levels of cyclin E1 expression were found in high-grade carcinomas than in low-grade carcinomas [20, 24]. We showed that cyclin E1 expression were higher in HCC patients than in the healthy participants. Notably, higher levels of cyclin E1 expression were associated with poor survival outcomes in HCC patients. Failure to respond to drug therapy may explain the fatalities in cancer therapy. Regorafenib and sorafenib are two major targeted drugs approved to treat HCC. It has been shown that cyclin E1 expression contributed to sorafenib resistance in HCC patients [16]. In this study, we found that cyclin E1 expression not only suppressed sorafenib induced apoptosis, but also compromised the therapeutic effects of regorafenib, which contributed to explaining why cyclin E1 expression leads to poor survival of HCC patients. Consistent with Hsu et al. [16], low dosage of sorafenib or regorafenib did not obviously suppress the expression of cyclin E1 (Fig. 2f), also suggested that its expression is an obstacle for these drugs. Furthermore, we found that the expression of cyclin E1 changes the transcription of Mcl-1 by enhancing STAT3 binding to the promoter of Mcl-1. Inhibition of cyclin E1 by CDK2 inhibitors abolishes the transcription of Mcl-1, and sensitizes HCC cells to regorafenib and sorafenib induced apoptosis (Additional file 5: Figure S5). Our in vivo data further suggested that the combination of regorafenib with cyclin E1 inhibition achieved better therapeutic effects by increasing HCC tumor apoptosis.

CDK inhibitors have reached the late research stages of some human cancer trials, with a potential for broader applicability since cell-cycle pathway aberrations are found across many different types of cancer [25]. Recently studies on developing CDK inhibitors to treat cancer have focused on palbociclib, abemaciclib, and ribociclib, which are selective CCND1/CDK4/6 inhibitors [26]. However, our data showed that CCND1, an important partner of CDK4/6, is not strongly correlated with HCC patient survival or anti-cancer drug response. Instead, we found that the expression of cyclin E1 played a more important role in HCC drug resistance. Our results suggest that FLA and DIN can strengthen the antitumor effects of sorafenib and regorafenib by suppressing the expression of Mcl-1. To date, FLA, a first-generation CDK inhibitor, is the most widely investigated. In the past few years, there have been more than 60 clinical trials involving FLA [27]. FLA can efficiently elicit G1 and G2 arrest, however can be cytotoxic in certain contexts [28]*.* In contrast, dinaciclib, as a second generation of CDK inhibitor, inhibits CDK2 specifically with less inhibitory effects on CDK4, CDK6, or CDK7. Cell-based assays [29] have shown that DIN is better able to suppress the phosphorylation of RB. Moreover, DIN can arrest the cell cycle in more than 100 cell lines from different tumor types and drive the regression of established solid tumors in various mouse models and clinical trials. Although the single-agent activity of FLA and DIN has been examined in many cancer types, treatments combining these inhibitors with other systemic therapies may enhance their antitumor effects [30]. Din and MK-2206 have previously been shown to be active against pancreatic adenocarcinoma [31] and FLA sensitizes HCC cells to TRAIL-induced apoptosis [32]. Our study supports the co-administration of CDK inhibitors and regorafenib or sorafenib for HCC therapy to improve their effectiveness. There is a concern that using general CDK inhibitors may cause off-target effects. Future clinical research on specific and multitargeted CDK inhibitors should help to determine a better clinical therapeutic index.

Mcl-1 expression is down-regulated by FLA and DIN [33, 34], while many other proteins are unaffected. Other researchers have proposed that this reflects the consequences of total transcription attenuation, since FLA and DIN also inhibited STAT3 [23, 35]. We were intrigued, however, by the fact that STAT3 regulates Mcl-1 transcription [36], which leads to our study. Our results showed, that the down-regulation of Mcl-1 may reflect decreased STAT3-regulated transcription due to cyclin E1 inhibitors, providing another potential mechanism to overcome drug resistant caused by abnormal Mcl-1 expression. However, the exact mechanism of STAT3 suppression by cyclin E1 inhibition is still unclear and need further efforts. Increasing evidence suggests that Mcl-1 plays an essential role in cancer cells, as Mcl-1 expression levels are often increased in cancer [37]. Decreased Mcl-1 levels can induce apoptosis even without other proapoptotic stimuli. Other than transcriptional modulation, Mcl-1 expression is also tightly controlled by post-transcriptional modification [22]. Regorafenib and sorafenib have been shown to promote the degradation of Mcl-1 by FBW7 [8]. Consistent with these studies, we detected significant inhibition of Mcl-1 expression by the combination of CDK2 inhibitors and regorafenib or sorafenib, indicating that transcriptional and post-transcriptional inhibition of Mcl-1 has synergic cancer killing effects. Moreover, inhibition of Mcl-1 at the transcriptional level by CDK2 inhibitors also provides a new opportunity to overcome drug resistance caused by the failure of Mcl-1 degradation, as FBW7 is frequently mutated in multiple cancers [8, 22, 38].

## Conclusions

In conclusion, our results indicate that in HCC cells cyclin E1 inhibition contributes to sorafenib-triggered apoptosis. Future studies that validate the value of cyclin E1 inhibitors are vital for predicting sorafenib’s effects. Among the potential challenges for combining cell-cycle regulators with established HCC clinical therapies is the identification of suitable biomarkers to evaluate treatment.

## Additional files

Additional file 1: **Figure S1.** CCNE1 expression levels are correlated to hepatocellular carcinoma cell sensitivity to regorafenib and sorafenib. A. Comparison of regorafenib and sorafenib IC50 in HCC cells with high and low CCNE1 expression. B. The summary of CCNE1 expression levels and regorafenib and sorafenib IC50 in different HCC cell lines. **, *P* < 0.05. (TIF 196 kb).

Additional file 2 **Figure S2.** CCNE1 expression suppressed the apoptosis induced by regorafenib or sorafenib. A. The cell viability of HepG2 cells transfected with the control or CCNE1 plasmid. B. Hoechst 33258 staining for apoptosis of HepG2 cells transfected with the control or CCNE1 plasmid and treated with 8 μM regorafenib or 5 μM sorafenib. C. A representative picture of the flow cytometry analysis of Annexin V/PI staining for apoptosis of Huh7 and HepG2 cells treated with 50 nM Din at the indicated time points. The flow cytometry was repeated for 3 times, and representative data were shown. *N* = 3 for A, B. *, *P* < 0.05, **, *P* < 0.05. (TIF 775 kb).

Additional file 3 **Figure S3.** Depletion of CCNE1 sensitized hepatocellular carcinoma cells to regorafenib and sorafenib. A. The cell viability of HepG2 cells transfected with the control or CCNE1 siRNAs. B. The expression of CCNE1 and cleaved caspase-3 in Huh7 cells transfected with the control or CCNE1 siRNAs treated with 8 μM regorafenib or 5 μM sorafenib. C. Hoechst 33258 staining for apoptosis of Huh7 cells transfected with the control or CCNE1 siRNAs treated with 8 μM regorafenib or 5 μM sorafenib. D. Hoechst 33258 staining for apoptosis of SK-HEP-1 cells treated with 50 nM Din (left) or 100 nM FLA (right) in combination with 8 μM regorafenib or 5 μM sorafenib. The western blots were repeated for 3 times, and representative data were shown. *N* = 3 for C, D. *, *P* < 0.05, **, *P* < 0.05. (TIF 423 kb).

Additional file 4 **Figure S4**. Mcl-1 expression suppressed the effect of CCNE1 inhibition. A. Hoechst 33258 staining for apoptosis of Huh7 cells transfected with the control or Mcl-1 plasmid and treated with 50 nM Din in combination with 5 μM sorafenib. B. Expression levels of cleaved caspase-3 and Mcl-1 in Huh7 cells transfected with the control or Mcl-1 plasmid and treated with 50 nM Din in combination with 5 μM sorafenib. The western blots and flow cytometry were repeated for 3 times, and representative data were shown. *N* = 3 for A. *, *P* < 0.05, **, *P* < 0.05. (TIF 254 kb).

Additional file 5 **Figure S5.** A summarized model of action. (TIF 300 kb).

## Data Availability

The datasets used and/or analysed during the current study are available from the corresponding author on reasonable request.

## Abbreviations

CCND: Cyclin D
CCNE1: Cyclin E1
CDK: Cyclin-dependent kinase
ChIP: Cell Signaling Technology
CHX: Cycloheximide
DIN: Inhibitors dinaciclib
DMSO: Dimethyl sulfoxide
FLA: Flavopiridol
HCC: Hepatocellular carcinoma
PI: Propidium iodide
TCGA: The cancer genome atlas
TUNEL: Transferase dUTP nick end labeling
