# Supplementary figures and images for: Inhibition of cyclin E1 sensitizes hepatocellular carcinoma cells to regorafenib by mcl-1 suppression
Source: Cell Commun Signal. 2019 Jul 26;17:85. doi: 10.1186/s12964-019-0398-3 (PMC6660968; doi:10.1186/s12964-019-0398-3)

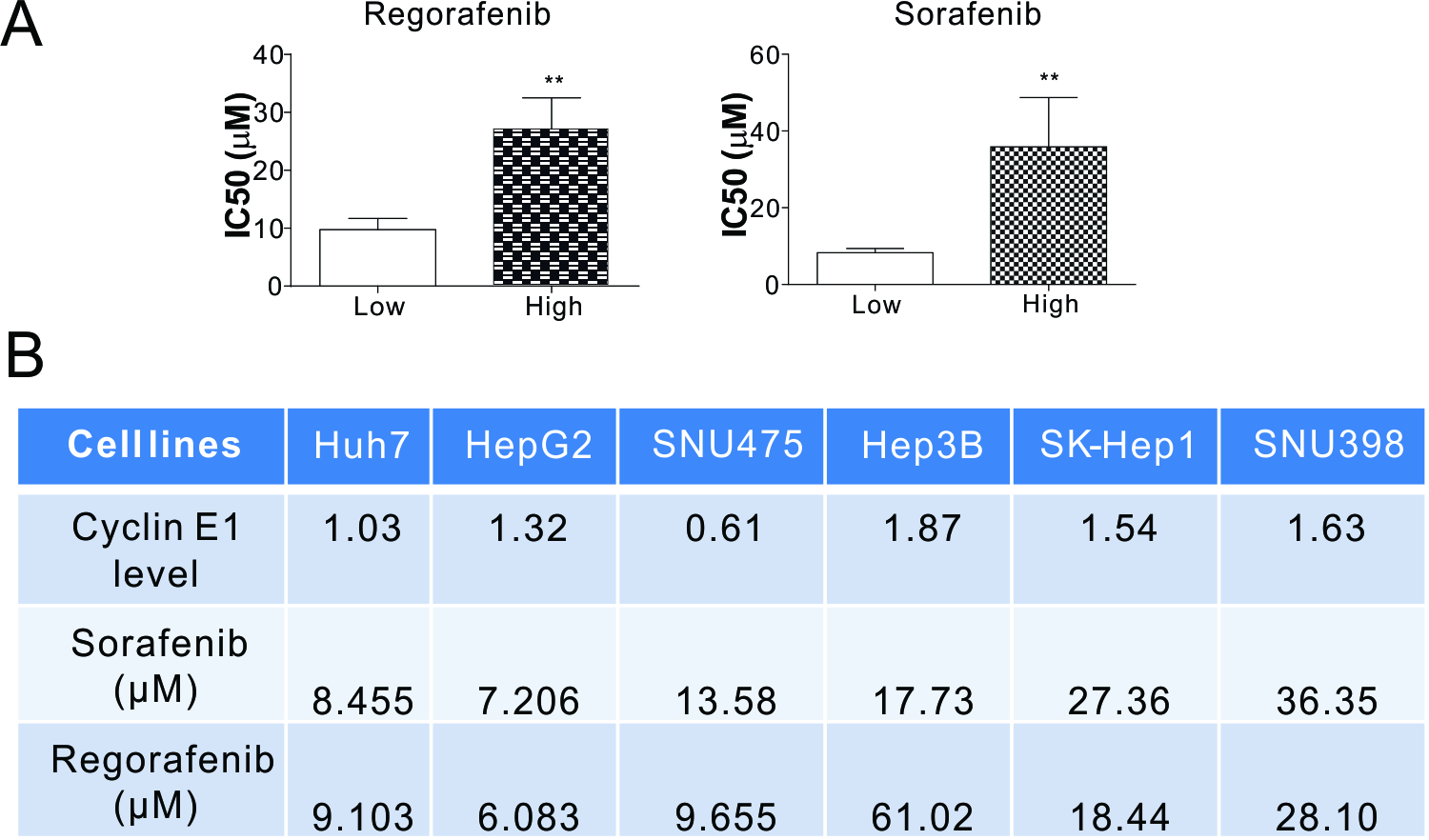

Supplement: Supplementary file 1 — Figure S1. CCNE1 expression levels are correlated to hepatocellular carcinoma cell sensitivity to regorafenib and sorafenib. A. Comparison of regorafenib and sorafenib IC50 in HCC cells with high and low CCNE1 expression. B. The summary of CCNE1 expression levels and regorafenib and sorafenib IC50 in different HCC cell lines. **, P < 0.05. (TIF 196 kb). [file 12964_2019_398_MOESM1_ESM.tif]

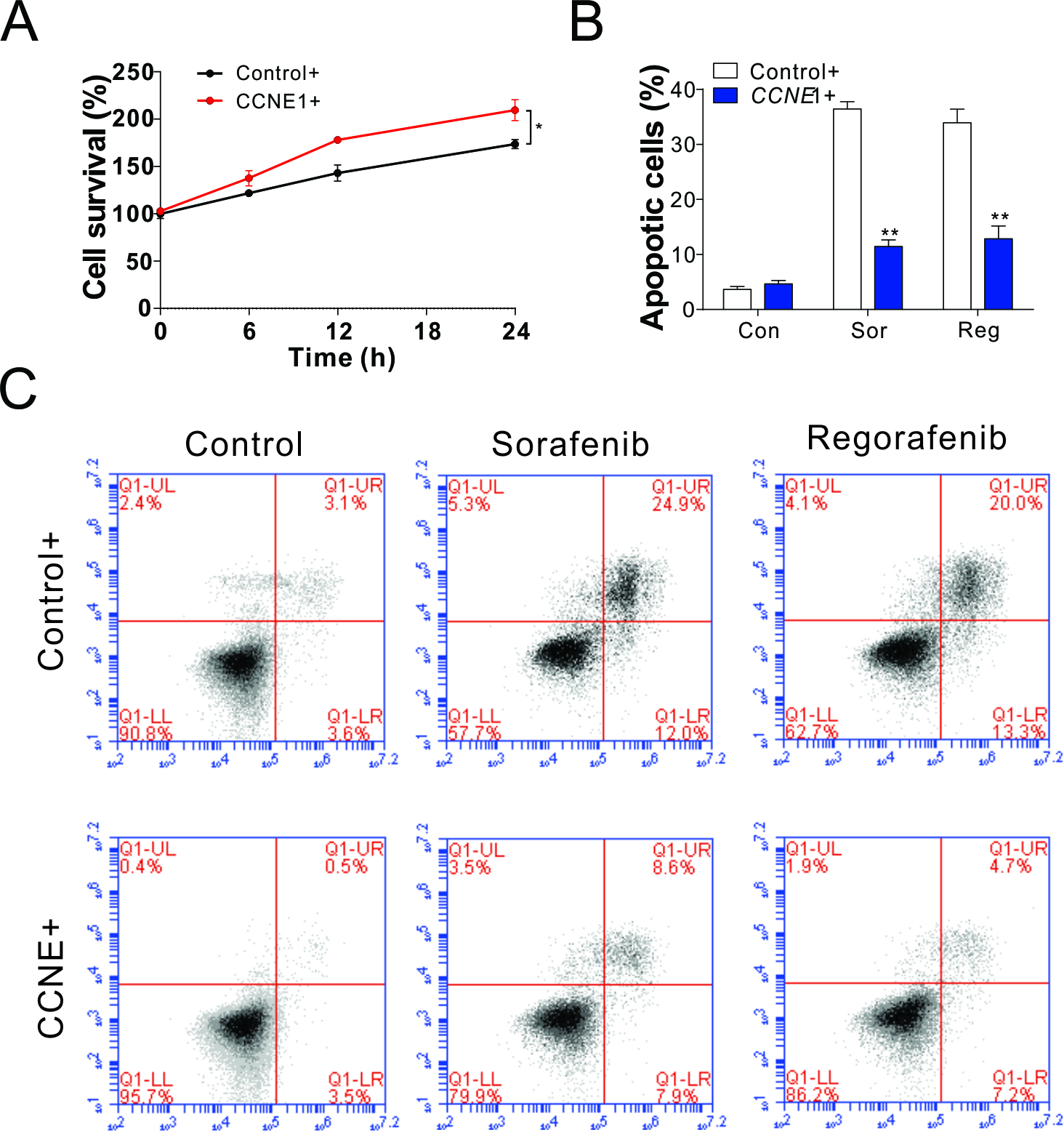

Supplement: Supplementary file 2 — Figure S2. CCNE1 expression suppressed the apoptosis induced by regorafenib or sorafenib. A. The cell viability of HepG2 cells transfected with the control or CCNE1 plasmid. B. Hoechst 33258 staining for apoptosis of HepG2 cells transfected with the control or CCNE1 plasmid and treated with 8 μM regorafenib or 5 μM sorafenib. C. A representative picture of the flow cytometry analysis of Annexin V/PI staining for apoptosis of Huh7 and HepG2 cells treated with 50 nM Din at the indicated time points. The flow cytometry was repeated for 3 times, and representative data were shown. N = 3 for A, B. *, P < 0.05, **, P < 0.05. (TIF 775 kb). [file 12964_2019_398_MOESM2_ESM.tif]

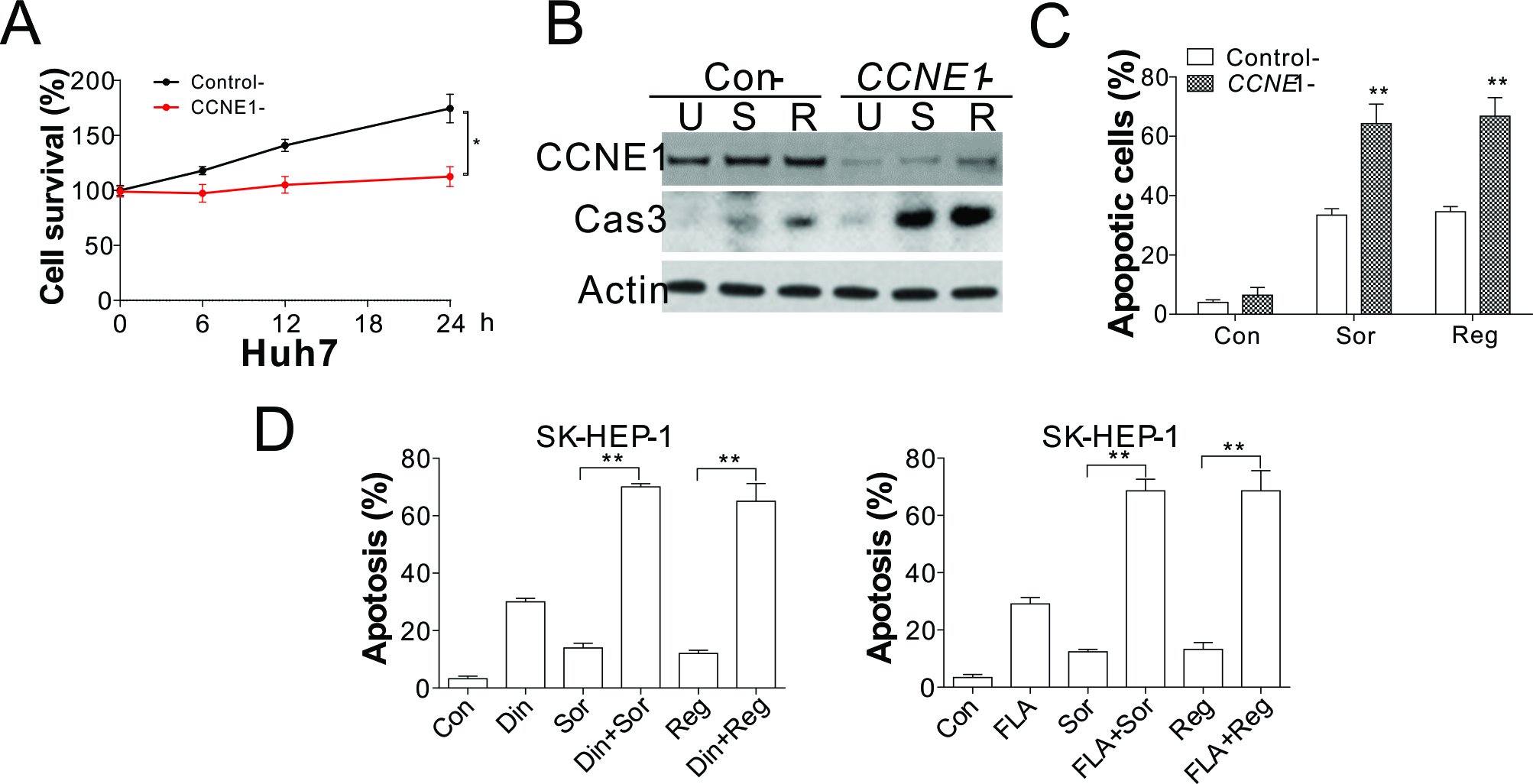

Supplement: Supplementary file 3 — Figure S3. Depletion of CCNE1 sensitized hepatocellular carcinoma cells to regorafenib and sorafenib. A. The cell viability of HepG2 cells transfected with the control or CCNE1 siRNAs. B. The expression of CCNE1 and cleaved caspase-3 in Huh7 cells transfected with the control or CCNE1 siRNAs treated with 8 μM regorafenib or 5 μM sorafenib. C. Hoechst 33258 staining for apoptosis of Huh7 cells transfected with the control or CCNE1 siRNAs treated with 8 μM regorafenib or 5 μM sorafenib. D. Hoechst 33258 staining for apoptosis of SK-HEP-1 cells treated with 50 nM Din (left) or 100 nM FLA (right) in combination with 8 μM regorafenib or 5 μM sorafenib. The western blots were repeated for 3 times, and representative data were shown. N = 3 for C, D. *, P < 0.05, **, P < 0.05. (TIF 423 kb). [file 12964_2019_398_MOESM3_ESM.tif]

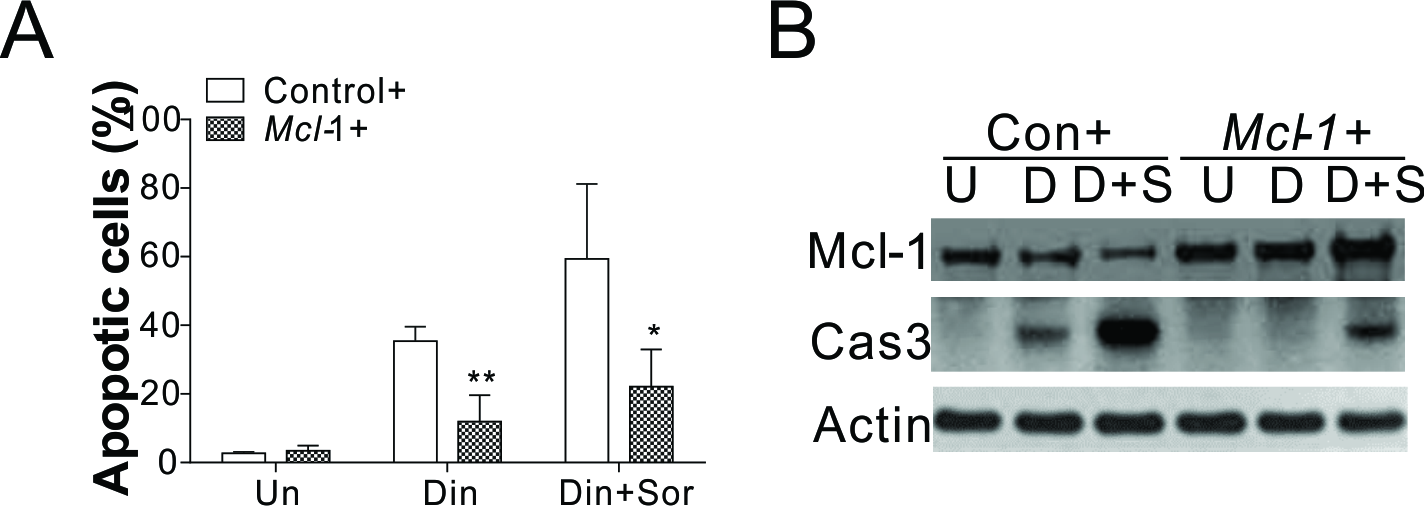

Supplement: Supplementary file 4 — Figure S4. Mcl-1 expression suppressed the effect of CCNE1 inhibition. A. Hoechst 33258 staining for apoptosis of Huh7 cells transfected with the control or Mcl-1 plasmid and treated with 50 nM Din in combination with 5 μM sorafenib. B. Expression levels of cleaved caspase-3 and Mcl-1 in Huh7 cells transfected with the control or Mcl-1 plasmid and treated with 50 nM Din in combination with 5 μM sorafenib. The western blots and flow cytometry were repeated for 3 times, and representative data were shown. N = 3 for A. *, P < 0.05, **, P < 0.05. (TIF 254 kb). [file 12964_2019_398_MOESM4_ESM.tif]

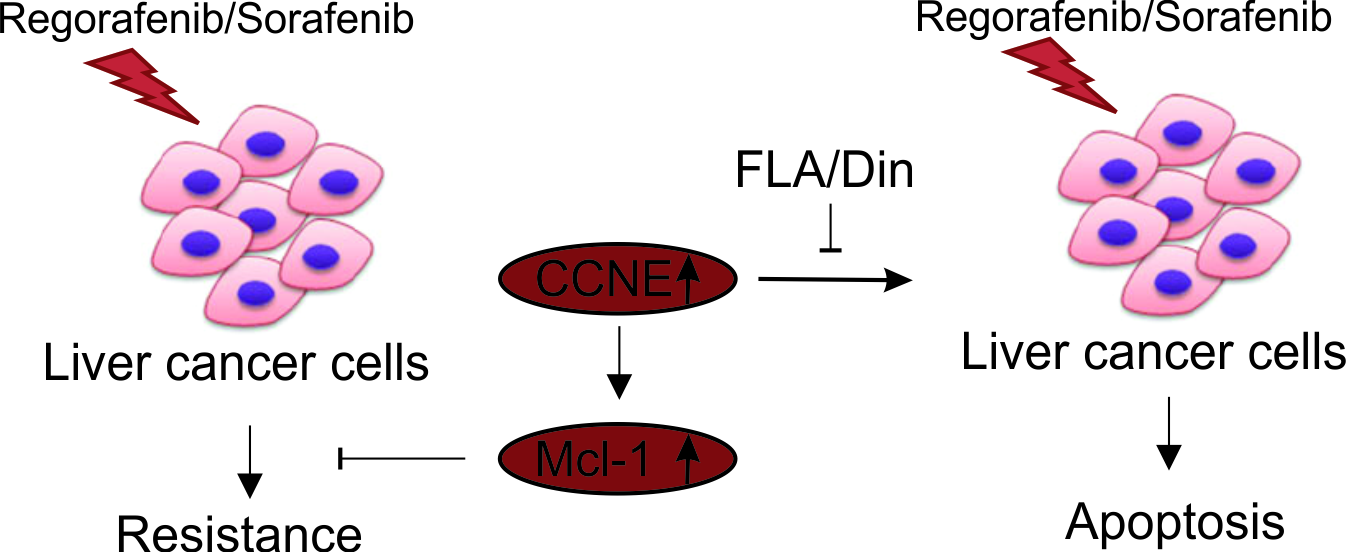

Supplement: Supplementary file 5 — Figure S5. A summarized model of action. (TIF 300 kb). [file 12964_2019_398_MOESM5_ESM.tif]
